# Interoception in individuals with autism spectrum disorder: a systematic literature review and meta-analysis

**DOI:** 10.3389/fpsyt.2025.1573263

**Published:** 2025-08-20

**Authors:** Marlene Klein, Michael Witthöft, Stefanie Maria Jungmann

**Affiliations:** ^1^ Department of Clinical Psychology, Psychotherapy and Experimental Psychopathology, Johannes Gutenberg University Mainz, Mainz, Germany; ^2^ Department of Clinical Psychology and Psychotherapy of Childhood and Adolescence, Johannes Gutenberg University Mainz, Mainz, Germany

**Keywords:** autism spectrum disorder, interoception, interoceptive accuracy, interoceptive awareness, interoceptive sensibility, meta-analysis

## Abstract

This systematic review and meta-analysis synthesize current research on interoception in individuals with autism spectrum disorder (ASD) across the lifespan. A total of 31 studies meeting stringent inclusion and quality criteria were identified, incorporating both descriptive and numerical analyses of cardiac interoceptive accuracy (cIA), interoceptive sensibility (IS), and interoceptive awareness (IAW) in children, adolescents, and adults with ASD. The meta-analysis of five adult studies using comparable assessment tools found no significant differences in cIA between adults with ASD and neurotypically developed (NTD) controls [*N* = 5; $\hat{\mu }$ = −.21 (SE = .11), *p* = .06]. Descriptive summaries of studies in children and adolescents, as well as those examining IS and IAW across age groups, revealed inconsistent findings—some studies reported reduced, increased, or similar interoceptive abilities in ASD compared to NTD. Methodological diversity, differences in measurement instruments, and variability in sample characteristics likely contribute to these inconsistencies. Moderator variables such as age, intelligence quotient (IQ), and comorbidities may influence interoceptive outcomes. Overall, the evidence indicates that ASD is not systematically associated with altered cIA, and the relationship between ASD and other interoceptive dimensions remains unclear. These findings underscore the need for more standardized methodologies and longitudinal research to clarify developmental trajectories and potential clinical implications of interoceptive processing in ASD. A deeper understanding of these mechanisms could lead interventions aimed at improving emotion regulation and social functioning in ASD.

**Systematic review registration:**

https://osf.io/f3ru4, identifier [doi.org/10.17605/OSF.IO/F3RU4].

## 1 Interoception and autism spectrum disorders

Interoception, the perception of bodily signals, comprises the processing of afferent neuronal signals (1) starting from organs, tissues, and physiological processes by the central nervous system (2). The perception of interoceptive signals is central for maintaining physiological states (3). Recent studies have found that interoceptive processes are related to psychopathological symptoms (Feldman 4–6), and altered interoception has also been suggested as a main vulnerability factor for psychopathology (7).

Autism spectrum disorders (ASDs) might have particularly close links to altered interoception due to their central characteristics such as hypo/hypersensitivity or unusual interest in sensory aspects of the environment (8) and alterations in emotion recognition, emotional processing, and social–emotional reactivity (1, 9). Earlier emotion theories assumed that physical changes are essential for emotions (10–12). Previous findings showed that cardiac interoception is related to emotional perception and regulation (13–17). A positive correlation was found between cardiac interoception and self-reported emotional arousal. Kever et al. (18) found positive correlations between cardiac interoception and the tendency to use the emotion regulation strategies reappraisal and suppression. In a network analysis, Yang et al. (17) found that higher interoceptive sensibility (IS) is associated with higher empathy and that individuals with higher autistic traits had lower levels of IS.

### 1.1 Conceptualization of interoception

Interoceptive processes can be described with different characteristics and divided into different dimensions (3). With regard to different dimensions, the conceptualization of interoception in terms of a three-component model and a 2 × 2 model are most familiar. The three-dimensional model of Garfinkel et al. (19) distinguishes the dimensions: interoceptive sensibility (IS), interoceptive accuracy (IA), and interoceptive awareness (IAW).

IS represents the self-assessment of how intensively bodily sensations can generally be perceived in comparison to other persons. In general, it is measured by self-rating questionnaires.

IA is a metric measure of how accurately individuals can perceive interoceptive signals. Regarding operationalization, many variants have been developed, which can be found in **Table 1**, such as the heartbeat tracking task (HTT) according to Schandry (20) or the heartbeat discrimination method (20–22). The HTT according to Schandry (20) is a performance task and requires a person to count his or her own heartbeats at a specified time interval. Discrimination procedures assess whether one or more rhythms of a presented external stimulus are synchronous or asynchronous to one’s own heartbeat (43).

**Table 1:** Common instruments to measure interoception.

Measuring method	Experimental/behavioral	Self-report questionnaires	Functional/structural magnetic resonance imaging	EEG
Relevant measuring instruments	Heartbeat tracking task: (Interoceptive accuracy) For adults, see (20); for children, see (21) Heartbeat discrimination task: (Interoceptive accuracy) (22, 23)Water load task: (Interoceptive accuracy) (24–27) Respiratory resistance load detection task: (Interoceptive accuracy) (24–27) Audiovisual simultaneity judgment task: (28) Cardiovisual simultaneity judgment task: (28)	eating disorder 1700 inventory-3 (EDI): subscale “interoceptive deficits” (Interoceptive sensitivity), (29, 30) BPQ: subscale “awareness” (Interoceptive sensitivity), (31, 32) Interoceptive Accuracy Scale (IAS): (confidence rating) (33) Interoceptive Confusion Questionnaire (ICQ): (confidence rating) (34) Multidimensional Assessment of Interoceptive Awareness (MAIA): (awareness) (35); BAQ: Body Awareness Questionnaire, (36)	Event-related potentials; resting-state functional connectivity (rsFC); Blood Oxygenation Level Dependent (BOLD) variability (rsBOLD), network connectivity at rest (networks of functionally connected brain regions); CONN-toolbox (fast Independent Component Analysis (ICA) algorithm); voxel analysis (37); Default Mode Network analysis, the temporal binding window analysis, Resting state functional neuroimaging, (28, 38–41)	Cortical representation of afferent cardiac signals; Heartbeat-evoked brain potentials (HEPs): (42)
Here are some exemplary instruments descriptions	Heartbeat tracking task: Participants are asked to count the number of heartbeats they feel in a given time interval without touching their body (i.e., not feeling the pulse in the forearm). The closer the participant’s reported number of heartbeats is to their actual number at the same time, the greater their cardiac interoceptive accuracy (cIA). Heartbeat discrimination task: Periodic external stimuli (e.g., tones, lights, or tactile stimuli) are presented and people have to decide whether these stimuli are synchronous or asynchronous with their own heartbeats.	MAIA: The MAIA is a questionnaire consisting of eight scales (Noticing, Not-Distracting, Not-Worrying, Attention Regulation, Emotional Awareness, Self-Regulation, Body Listening, and Trusting) to assess self-perceptions of interoceptive body awareness.	Temporal binding windows: Testing, for example, the cardiovisual synchrony in Stimulus Onset Asynchrony (SOA) independence.	

IAW is reflected in participants’ awareness of their performance in the perception of specific signals in a given time period, such as a test session (19). IAW estimates the amount of agreement regarding measured accuracy of performance tasks and self-assessed assurance in the tested ability.

In addition to this three-dimensional model, Murphy et al. (44) proposed a four-dimensional model, a 2 × 2 scheme resulting in four fields linked in lines and rows. The four-field scheme distinguishes interoceptive abilities on the basis of two factors: What is measured (accuracy versus attention) and how it is measured (objective performance versus self-report). Measurements play an important role in the context of this review. Differences in results could be caused by the optimalization of interoception. Therefore, **Table 1** summarizes the common measurement instruments used to assess interoceptive processes.

### 1.2 Interoception and emotion in ASD

The relationship between interoceptive mechanisms and emotional processing in ASD is grounded in neurobiological and behavioral evidence, with recent studies clarifying both structural and functional correlates. The somatic marker hypothesis (45) posits that interoceptive signals influence decision-making by linking physiological states of emotions to cognitive evaluations of decisions, while predictive coding models suggest that emotions arise from integration of predicted and actual bodily states (46). In ASD, attenuated precision-weighting of interoceptive prediction errors may disrupt this loop, leading to difficulties in emotion recognition and regulation.

#### 1.2.1 Neurochemical and neural substrates

Neuroimaging studies reveal that the anterior insular, a hub for interoceptive-affective integration, exhibits altered glutamate and gamma-aminobutyric acid (GABA) concentrations in ASD. Elevated glutamate levels in the left insular correlate with both alexithymia (difficulty identifying emotions) and heightened awareness of autonomic reactivity, suggesting hyperexcitability in interoceptive networks (47). Conversely, increased GABA in the anterior cingulate cortex is selectively associated with alexithymia, potentially reflecting compensatory inhibition of emotional arousal. These findings align with the anterior insular’s hypoactivation during social–emotional tasks in ASD (Cohen’s *d* = −0.72) and its reduced functional connectivity with the ACC, which predicts interoceptive confusion (48).

#### 1.2.2 Interoceptive confusion and alexithymia

Approximately 50% of individuals with ASD exhibit comorbid alexithymia, mediated by interoceptive deficits. The Interoception Sensory Questionnaire (ISQ), validated in autistic adults, demonstrates that 74% report significant interoceptive confusion unless bodily signals are extreme (48). This “alexisomia” (impaired somatic awareness) correlates strongly with Toronto Alexithymia Scale (TAS-20) scores (*r* = 0.76), particularly the Difficulty Identifying Feelings subscale, which mediates 62% of emotional clarity deficits in ASD ( $\text{Δ}R^{2}=0.38$ ). Qualitative data further reveal that low IA predicts reliance on maladaptive regulation strategies (e.g., suppression and rumination) and passive coping mechanisms, such as external cue dependence (49).

#### 1.2.3 Methodological and clinical implications

Critically, traditional measures like the heartbeat counting task show limited reliability in ASD populations due to reliance on top-down estimation rather than genuine interoceptive perception (47). Self-report tools like the ISQ and Multidimensional Assessment of Interoceptive Awareness (MAIA) better capture the lived experience of interoceptive confusion and its emotional consequences (49). Clinically, interventions targeting IAW (e.g., mindfulness and biofeedback) show promise, with randomized trials reporting medium effect size improvements in emotional granularity (Hedges’ *g* = 0.63). Neurofeedback protocols enhancing insular–anterior cingulate cortex connectivity (*η^2p^* =0.27) may further normalize interoceptive–emotional integration.

By integrating predictive coding frameworks with neurochemical and behavioral evidence, this model elucidates how interoceptive dysfunction in ASD embodied emotional representations, offering actionable targets for therapeutic intervention.

### 1.3 Aims of the present review

Exteroception is part of the diagnosis criteria in DSM 5 (50), but interoception being included as part of it is still in discussion. Owing to the importance of interoceptive processes and the above-mentioned overlap between interoception and ASD, a number of studies on the relationship between interoception and ASD have been published in recent years (17, 51–53). In 2016, a review on interoception in ASD (54) identified five studies with different methodological approaches [functional magnetic resonance imaging (fMRI) during interoceptive task, qualitatively questionnaires, other questionnaires, and Schandry task], which were qualitatively described (due to the low number and heterogeneity at that time) and summarized. The authors concluded that altered interoception in ASD can be presumed, but more details need to be investigated. Proff et al. (55) and Williams et al. (56) reviewed differences in individuals with ASD and neurotypically developed (NTD) individuals, but were less specific (e.g., considered exteroception as well as interoception). They did not analyze the existing results statistically in a meta-analysis. The narrative review of Loureiro et al. (57) covers the existing literature on the behavioral and neurophysiological aspects of interoception in individuals with ASD, highlighting variability and underlying mechanisms but also do not statistically consolidate the findings. They hypothesize on neurotypical and neurobiological reasons for differences between NTD and ASD. In addition, we aimed to statistically validate the comparison and found (see below) no significant results in the meta-analysis. Loureiro et al. (57) suggest that measurement issues could be compensated by including multiple measurements. Inconsistency in data would be related to the chosen measurement tools. As these three narrative reviews in particular illustrate, there is a great deal of research interest in this area. What is currently still missing is a systematic, broader overview (in terms of methods and age) and, in particular, a statistical summary of the results in the sense of a meta-analysis. By including a larger number of studies with a bigger range of age and applying careful inclusion criteria and quality ratings, this systematic overview and meta-analysis across the lifespan tries to address previous research heterogeneity. Considering more measurement tools through showing results of more studies using different tools to offer a detailed and statistically robust summary of available evidence aims to compensate for issues of specific tools. There needs to be a clear distinction between systematically reviewed literature results and statistically meta-analyzed evidence.

## 2 Method

### 2.1 Literature search and data collection

Following the PRISMA 2020 guidelines, a systematic literature search was conducted using the electronic databases PubMed, PsycINFO, and Web of Science. The initial search in December 2020 included keywords related to interoception and autism spectrum disorder (ASD): *(“interoception” OR “interoceptive” OR “heartbeat detection” OR “heartbeat perception” OR “heart rate detection” OR “heart rate perception” OR “cardiac detection” OR “cardiac perception”) AND (“autism” OR “autistic”)*.

In order to update and qualitatively test the search, a second search process was started in June 2021 using software-based MeSH words and truncations following the Yale MeSH Analyzer on https://mesh.med.yale.edu/. The search update was performed according to the PICO guidelines for literature searches (**P**opulation: ASD; **I**ntervention/Exposure: Interoceptive measures; **C**omparison: Neurotypical Controls; **O**utcomes: Interoceptive Accuracy/Sensibility/Awareness). The search-syntax was: (“pervasive developmental disorder*”[Title/Abstract] OR “kanner*”[Title/Abstract] OR “autism*”[Title/Abstract] OR “autist*”[Title/Abstract] OR “asperger*”[Title/Abstract] OR “ASD”[Title/Abstract] OR “Autistic Disorder”[MeSH Terms]) AND (“alliesthesia*”[Title/Abstract] OR “interocept*”[Title/Abstract] OR “Interoception” [MeSH Terms]) (58).

### 2.2 Data evaluation: inclusion and exclusion criteria

Animal experiments were excluded. Inclusion criteria were as follows: (i) a focus on interoceptive processes; (ii) investigation of diagnosis of ASD (8); (iii) published research article; (iv) empirical status; (v) peer reviewed; and (vi) full text written in English or German. There were no age restrictions, and the studies in this review are structured (if possible) into adult and child/adolescent studies.

When studies seem to have utilized the same dataset, the redundant data were excluded.

### 2.3 Study selection and synthesis process

#### 2.3.1 Data analysis

**Figure 1:**
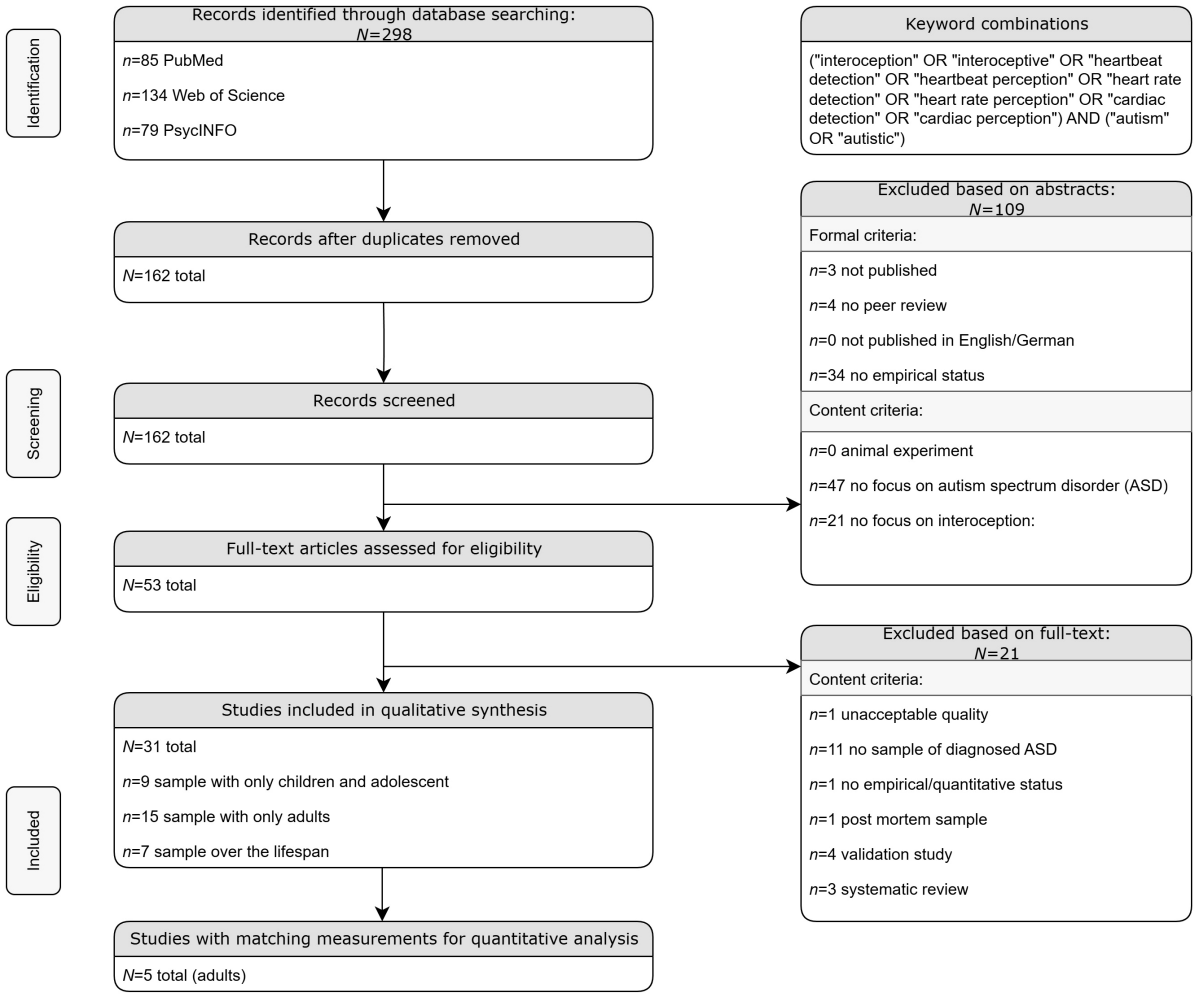
Flow Chart.

In 2021, the preparation and project planning began. The study design got preregistered at the Open Science Framework (OSF) https://osf.io/f3ru4 in December 2021. **Figure 1** shows the flow of information and different steps in identification and verification of relevant articles. After the duplicates were removed and the articles were assessed according to the inclusion and exclusion criteria, quality ratings were conducted. The papers were reviewed by three raters in a full text analysis, based on systematically elaborated criteria (59), which have been slightly adapted for the current requirements. The following criteria were used: (a) consecutive sampling; (b) sufficient and comprehensible presentation of the inclusion and exclusion criteria; (c) sample size, based on effect size and power calculation, in line with commonly used conventions (59): very small, <15 participants (power < 0.80 to identify a very large effect size of Cohen’s *d* = 0.11); small, <26 participants (power < 0.80 to identify a large effect size of *d* = 0.8); moderate, 26–63 participants (power ≥ 0.80 to identify a large effect size of *d* = 0.8); and large, ≥64 participants in each group (power ≥ 0.80 to identify a medium effect size of *d* = 0.5); (d) comparison group matched by age and gender; (e) standardized (behavioral tasks) or validated (self-reports) measures; and (f) use of inferential statistics. The overall study quality was calculated using the proportion of positive matching ratings and consideration of the sample size. The quality was assessed as high (with ≥80% matches of positive criteria and a large sample size), medium (with 50%–79% positive matches and at least a moderate sample size or ≥50%), low (with 20%–49% judging “yes” or a low sample size), or unacceptable (with ≤20% or a very low sample size) (59).

Of the 31 studies that met the inclusion criteria, 5 (16.1%) were rated to be of high quality (31, 52, 53, 60, 61); 13 (41.9%) were rated to be of medium quality (17, 51, 62–72); and 13 (41.9%) were rated to be of low quality, mainly due to low sample size (38, 60, 73, 74; 19, 28, 75–82). We achieved an agreement between the three raters of Cohen’s kappa = 0.82. The individual quality ratings for each article are depicted in **Supplementary Material 1**.

Not all studies were suitable for a quantitative data analysis or meta-analytic approach. A modest number of samples using comparable measurement instruments and reporting complete results or providing sufficient additional material was found only in the adult studies. Of the 15 adult studies, 10 used nearly the same heartbeat perception task and 5 of them reported or e-mailed us the necessary statistical results.

With the small number of studies in childhood and adolescence (*N* = 8), there is too much heterogeneity in design, measurement instruments, and comparison groups for a quantitative analysis; therefore, the collected studies for children/adolescents can only be qualitatively summarized in a narrative review.

#### 2.3.2 Method of quantitative analysis

First, statistical heterogeneity tests were used (*Q* and *I* ^2^) to examine the heterogeneity/homogeneity of the studies (83). The analysis follows the descriptions of Hunter and Schmidt (84) using the R package “metafor” (version 2.4-0) as recommended by Viechtbauer (85).

### 2.4 Risk of bias assessment and PRISMA compliance

In accordance to the PRISMA 2020 guidelines, we implemented a comprehensive risk of bias assessment for all included studies. Three independent reviewers evaluated each study using a modified version of the Newcastle–Ottawa Scale for observational studies, focusing on three domains: (1) selection of study groups, (2) comparability of groups, and (3) ascertainment of exposure and outcomes. Discrepancies in ratings were resolved through discussions or consultation with a fourth reviewer. Studies with a Newcastle–Ottawa score below 4 were excluded from quantitative synthesis to minimize the impact of low-quality evidence.

To further address potential sources of bias across studies, we systematically assessed and reported heterogeneity using several statistical measures. Specifically, we calculated Cochran’s *Q* statistic to test for the presence of heterogeneity, the *I* ^2^ statistic to quantify the proportion of total variation due to heterogeneity rather than chance (with *I* ^2^≥ 50% indicating moderate to substantial heterogeneity), and $\tau^{2}$ to estimate variance between studies. These measures were reported in both the Methods and the Results sections to transparently communicate the degree of heterogeneity and inform about the choice of meta-analytic models.

We also conducted sensitivity analyses, including leave-one-out analyses (e.g., by age group and measurement instrument), to determine the robustness of our findings and to identify any influential studies or subgroups that contributed disproportionality to heterogeneity or potential bias.

Visual assessment of heterogeneity and potential publication bias was conducted using standardized residual plots and funnel plots. Funnel plots display effect sizes against their standard errors, with asymmetry potentially indicating publication bias or small-study effects. Publication bias was evaluated using funnel plot asymmetry and Egger’s regression test for meta-analyses with more than 10 studies. Small-study effects were addressed by excluding studies with a sample size below 20 from the quantitative synthesis.

A completed PRISMA 2020 checklist is provided in **Supplementary Material 2**, detailing our adherence to all relevant items, including explicit reporting of risk of bias assessment methods (item 8), heterogeneity measures (item 13d-f), and sensitivity analyses (item 13f).

## 3 Results of the literature search

### 3.1 Summary overview regarding ASD in childhood and adolescence

Because of the heterogeneity of the studies, no meta-analysis was possible; therefore, the results are qualitatively reported. The original Schandry task was developed and validated for adults. When the cardiac perception was tested in children and adolescents, adjustments were made to adapt it to their age. The studies for children and adolescents each included different age ranges. Accordingly, the language of the instructions was also adapted to the respective age. Furthermore, the adaptations differed in the duration of the trials to the presumed attention span and the number range (numerical development) of the children. The researchers chose different sequences, which they then extrapolated. Overall, too different heart rate perception tests were carried out, too different age ranges were examined, and, above all, no uniform statistical parameters were reported for a meta-analytical investigation to be possible. Information about the samples and an overview of the main results are listed in **Table 2**.

**Table 2:** Results of children and adolescents.

Study	*N*; group	Age: M (SD)	*N* females	Age range	Key findings heartbeat perception (cIA)	Key sensibility findings (via BPQ, MAIA, or SCR)	Key awareness findings
(38)	*N* (ASD) = 14 *N* (NTD) = 15	M (SD) = 15.79 (1.93) M (SD) = 15.95 (1.65)	4 2	12–20 12–20	–	–	–
(80)	*N* (ASD) = 21 *N* (NTD) = 21	M (SD) = 12.28 (2.8) M (SD) = 11.52 (2.5)	2 4	8–17 8–17	ASD = NTD (tracking)	–	–
(31)	*N* (ASD) = 30 *N* (NTD) = 30	M (SD) = 12.5 (2.9) M (SD) = 11.9 (3)	Not reported Not reported	6–18 6–18	ASD < NTD* (tracking) ASD = NTD (discrimination)	ASD = NTD (BPQ)	ASD < NTD*
(67)	*N* (ASD) = 21 *N* (NTD) = 21	M (SD) = 12.95 (1.49) M (SD) = 12.70 (1.17)	5 6	10–16 10–16	ASD < NTD*	–	–
(68)	*N* (ASD) = 49 *N* (NTD) = 0	M (SD) = 12.80 (2.91)	9	6–19	–	–	–
(70)	*N* (ASD) = 61 *N* (NTD) = 62	M (SD) = 13.46 (1.77) M (SD) = 13.52 (1.57)	54 60	11–18 11–18	–	ASD < NTD*	ASD = NTD
(69)	*N* (ASD) = 55 *N* (NTD) = 44	M (SD) = 13.27 (2.75) M (SD) = 13.18 (3.39)	20 21	7–19 6–18	ASD = NTD (tracking)	ASD < NTD* (BPQ)	–
(62)	*N* (ASD) = 35 *N* (NTD) = 40	M (SD) = 11.95 (2.19) M (SD) = 11.86 (2.17)	7 7	8–17 8–17	–	ASD = NTD (BPQ-VSF)	–
**(****17****)**	*N (*ASD*) =* 30 *N* (NTD) = 0	M (SD) = 8.63 (2.01)	*7*	6–13	ASD < NTD (EIAT)	–	–

*: α< 0.05.

**: α< 0.01.

Schauder et al. (80) compared children and adolescents with ASD and NTD in the HTT (20). There was no significant group difference in cardiac interoceptive accuracy (cIA). Comparing the performance of long and short intervals (the HTT includes long and short periods of perceptual tasks) revealed differences between groups. There was a difference between short and long intervals within the NTD sample (*p* = 0.001, *d* = 0.150), while there was no difference within the sample of individuals with ASD (*p* = 0.525, *d* = 0.605).

Palser et al. (31) found a significant difference between the ASD and NTD samples in cIA, when measured with the HTT according to Schandry (20) (the ASD sample showed significantly lower cIA, *p* = 0.001, *d = −*0.109). They found no significant differences between the groups in cIA (*p* = 0.782, *d* = 0.046) when using heartbeat discrimination according to Katkin and Deroo (23) and in IS (*p* = 0.847, *d* = 0.050 via PBQ, 32). When ASD is viewed dimensionally (severity via ADOS-2), no significant correlations were shown between autism severity and cIA (*r*_s_ ≤ 1.161, *p*s ≥ 0.40). Regarding IAW, the ASD sample showed significantly more confidence in assessing their discrimination performance (*p* = 0.006, *η^2^_p_=*0.123).

Nicholson et al. (67) discovered a significantly lower cIA (Schandry (20)) in children and adolescents with ASD compared to NTD (*p* = 0.024, *η^2^_p_* = 0.120).

Pickard et al. (70) identified no significant group differences (Wilcoxon test statistic, *r =* 0.14) in cIA according to Schandry (20). Individuals with ASD reported significantly reduced IS (*d* = 0.58) and IAW (Wilcoxon test statistic, *r =* 0.21) compared to NTD.

Palser et al. (69) found no significant group difference (*B* = 0.170, *p* = 0.221) for cIA after Schandry (20). Individuals with ASD showed significantly lower IS (*B* = 0.199, *p* = 0.043) (BPQ, 32) compared to NTD.

Palser et al. (68) considered in regression analyses the dependent variables total ASD symptom severity, social–affective symptom severity, and repetitive–restrictive behaviors. For overall ASD symptom severity, cIA (20, and heartbeat discrimination task, 22, 86) was not a significant predictor (*B* ≤ 0.071, *p* ≥ 0.596); confidence in performance was shown to be a significant predictor in each case (discrimination: *B* = 0.449, *p* = 0.011, tracking: *B* =-0.405, *p* = 0.049). Regarding the social–affective symptoms of ASD, only confidence in the heartbeat discrimination task was shown to be a significant predictor (*B* = 0.458, *p* = 0.011). Repetitive–restrictive behavior was significantly correlated only with cIA, measured with the discrimination task (*B* = 0.399, *p* = 0.013).

Butera et al. (62) found no significant difference in IS [Body Perception Questionnaire: BPQ—Very Short Form (32, 87), *p* = 0.891, *BF* = 4.501)] between individuals with ASD and NTD.

Yang et al. (17) measured symptoms of ASD and attention-deficit hyperactivity disorder (ADHD) indicated by a parent report and IA (Eye-Tracking Interoceptive Accuracy Task). They investigated three samples: (1) children with ASD (*N* = 30), (2) children with ASD and ADHD (*N* = 20), and (3) NTD (*N* = 63) with high and low levels of autistic traits. Group differences in IA between ASD and NTD children were significant. ASD children with and without comorbid ADHD showed significantly lower cIA (20) than NTD (*p* < 0.001), with no significant difference between the two clinical groups (Groups 1 and 2: *p* = 0.27). cIA was lower in NTD children with high autistic traits compared to children with lower autistic traits. There were negative correlations between cIA and ASD severity (*r = −*0.326, *p* < 0.01) as well as ADHD symptoms (*r =* −0.425, *p* < 0.01).

#### 3.1.1 Neurophysiological findings

Ebisch et al. (38) found differences in functional connectivity of brain regions. The connectivity of the insular cortex with the amygdala and somatosensory regions was significantly reduced in individuals with high functional ASD compared with NTD. Further information about the described papers is shown in **Table 2**.

Insular connectivity (72): Reduced functional connectivity between the insular cortex and amygdala/somatosensory regions was observed in ASD compared to NTD, suggesting altered integration of interoceptive and emotional signals.

Thalamic connectivity (72): ASD samples showed stronger thalamic connectivity with somatosensory, motor, auditory, and interoceptive cortices, potentially reflecting compensatory mechanisms for atypical sensory processing.

### 3.2 Summary review regarding ASD in adults

Shah et al. (81) found no significant group difference in cIA (20) in adults with ASD vs. NTD (*p* = 0.46, *d* = 0.23).

Shah et al. (71) also showed that ASD and NTD did not differ significantly (*p* = 0.43, *d* = 0.26) in cIA (20). Among individuals with ASD and in the overall sample, no significant correlations between ASD severity and cIA were found (*r* ≤ −0.22, *p* ≥ 0.23). Owing to the same recruitment pathway and similar sample sizes and sociodemographics, Shah et al. (81) and Shah, Hall et al. (71) seem to have used almost the same sample (*N* = 20 and 19 individuals with ASD, respectively).

Garfinkel et al. (88) found a significant group difference in cIA, measured with the HTT (*p* = 0.001, *d* = 1.10, 20), but no significant group difference in the heartbeat discrimination task (*p* = 0.28, *d* = 0.35). Individuals with ASD scored significantly higher regarding IS [*p* < 0.001, *d* =-2.02, BPQ, Porges (32)], but there was no group effect concerning IAW (*p* = 0.57, *d* = −0.19). They calculated via Interoceptive Trait Prediction Error (difference between objective cIA and IS) whether the groups over- or underestimated their performance. Individuals with ASD overestimated IS, in which they scored higher relative to cIA. NTD underestimated their IS relative to the measured cIA. This ITPE significantly differed between the two groups (*p* < 0.001, *d* = −2.49).

Nicholson et al. (79) found no significant difference (*p* = 0.53, *d* = 0.13) between individuals in the ASD and NTD groups regarding cIA according to Schandry (20).

In the study of Mul et al. (66), individuals with ASD showed significantly reduced cIA (*p* = 0.04, *d* = 0.58), but only when measured with the Schandry (20) mental tracking task. In contrast, there was no significant difference (*p* = 0.39, *d* = 0.24) when using the heartbeat discrimination task (22, 86). Individuals with ASD reported significantly lower IS (*p* = 0.02, *d* = 0.70) (Multidimensional Assessment of IAW: MAIA, 35). IS (*r =* −0.57, *p* < 0.001) and cIA via heartbeat detection (*r =* −0.29, *p* ≤ 0.05; not via heartbeat tracking *r = −*0.04, *p* > 0.05) showed significant negative correlations with autistic traits [Autism Questionnaire (AQ), 89].

Nicholson et al. (67) found no significant group difference in cIA according to Schandry (20) (*p* = 0.86, *d* = 0.06) or respiratory IA (Blow Comparison Task) (*p* = 0.62, *d* = 0.15) between those with ASD and NTD.

Fiene and Brownlow (75) measured IS with the Body Awareness Questionnaire (BAQ; 36) and the Thirst Awareness Scale [TAS; developed within this paper by the authors (75)]. The ASD sample showed significantly lower body (*p* < 0.001, *d* = 1.26) and thirst awareness (*p* < 0.001, *d* = −1.02) in the self-assessment compared to the NTD sample.

Mulcahy et al. (61) found no main effect in cIA by group (*p* = 0.15, 20, 22, 86). The cIA did not appear to consistently impact how well individuals with ASD could interpret emotional tone of voice. There was also no main effect of MAIA total score (*p* = 0.15). They found a main effect of IAW on the discrimination task (*p* = 0.05).

Mul et al. (65) reported inconsistent findings regarding group differences in IS. With regard to the subscale attention regulation of the MAIA, persons with ASD showed significantly lower scores than NTD (*p* < 0.007), but no significant group difference was found for the subscale noticing.

Bird et al. (74) showed that those with ASD and NTD rated the valence of pain in pleasant–unpleasant ratings at low pain level significantly differently (ASD approximately at zero, NTD positive; *p* ≤ 0.02), but not for the high pain condition (*p* ≥ 0.43).

Gu et al. (77) measured cortical response on fMRI during an empathy-for-pain task and skin conductance response (SCR). The ASD sample showed significantly increased SCR related to empathic pain (*p* = 0.05), together with increased neuronal activity in the anterior insular cortex. They found significantly decreased behavioral empathetic pain discriminability in the ASD sample (*p* < 0.01). The overall event evoked SCR rate was lower in the ASD sample.

Gu et al. (78) analyzed pain perception as well as pain expectancy, fMRI, and SCR. The level of pain caused by electrical density, which was rated as moderately painful, was significantly different in the tested sample groups, and the individuals with ASD chose a lower electrical intensity than the NTD sample group (*p* = 0.016). While there was no difference for the tested groups in insular activation during anticipation of pain and perception of pain, the individuals with ASD had a higher activation in the dorsal and rostral anterior cingulate cortex when anticipating pain. There was a correlation between SCR and the left anterior insular while pain was expected across all participants (*r* = 0.44, *p* = 0.015).

Nisticò et al. (51) investigated sensory sensitivity (via the Sensory Perception Quotient) in ASD. Increased tactile hypersensitivity was found to be associated with functional weakness (OR = 0.74, *p* = 0.033) and paresthesia (OR = 0.753, *p* = 0.019).

#### 3.2.2 Neurophysiological findings in adults

During empathy-for-pain tasks, individuals with ASWD exhibited increased anterior insular cortex activation alongside elevated skin conductance (SCR), despite reduced behavioral pain discrimination (77).

Bernhardt et al. (73) focused on structural brain networks that are associated with socio-cognitive and socio-affective functions in ASD. Compared to NTD, individuals with ASD showed significantly lower covariance in the dorsomedial prefrontal cortex and temporo-parietal junction networks (both assumed to be related to Theory of Mind), in contrast to results regarding the fronto-insular cortex networks (assumed to be related to interoception and empathy).

In the study of Gaigg et al. (76), participants (ASD and NTD) saw 70 emotion-triggering images and were asked to rate their subjectively perceived arousal and the valence. The researchers also measured arousal objectively by SCR. There was no significant difference regarding SCR between the two groups (*p* = 0.59). The subjectively perceived arousal and the objectively measured arousal by SCR correlated significantly in ASD (*r* = 0.51, *p* ≤ 0.001) and NTD (*r* = 0.55, *p* ≤ 0.001), when controlling for valence ratings.

### 3.3 Summary review regarding ASD over the lifespan (mixed age samples)

The studies with results over the lifespan are shown in **Table 3**.

**Table 3:** Results over the lifespan.

Study	*N*; group	Age: M (SD)	*N* females	Age range	Key findings heartbeat perception (cIA)	Key sensibility findings (via BPQ, MAIA, or SCR)	Key awareness findings
(64)	*N* (ASD) = 56 *N* (NTD) = 58	M (SD) = 29.63 (11.29) M (SD) = 31.47 (9.51)	18 21	8–54 8–54	ASD = NTD (tracking)	–	–
(82)	*N* (ASD) = 25 *N* (NTD) = 26	M (SD) = Not reported M (SD) = Not reported		16–61 18–50	–	–	–
(28)	N (ASD) = 23 N (NTD) = 31	M (SD) = 22.0 (4.24) M (SD) = Not reported	8 Not reported	14–29 Not reported	Only control task for ASD sample (tracking)	–	–
(52)	*N* (ASD) = 565 *N* (NTD) = 602 *N* (ASD) = 91 *N* (NTD) = 233	M (SD) = 15.3 (6.8) M (SD) = 15.5 (6.4) M (SD) = 15.5 (9.1) M (SD) = 14.2 (7.5)	0 0 91 233	7–40 7–40 7–40 7–40	–	–	–
(60)	*N* (ASD) = 46 *N* (NTD) = 54	M (SD) = 19.43 (10.68) M (SD) = 21.43 (10.41)	14 19	8–54 8–53	ASD = NTD (tracking)		
(63)	*N* (ASD) = 51 *N* (suspected ASD) = 32 *N* (NTD) = 119	M (SD) = 33.9 (13.5) M (SD) = 36.1 (13.5) M (SD) = 28.9 (12.0)	34 22 11	16–62 19–67 17–64	–	ASD = NSD (BAQ; Shields)	–
(52)	*N* (ASD) = 76	M (SD) = 17.91 (1.93)	32	16–25	–	–	–

NTD, neurotypically developed; BPQ-VSF, Body Perception Questionnaire Very Short Form; BAQ, Body Awareness Questionnaire; EIAT, the eye-tracking interoceptive accuracy task; ASD, autism spectrum disorder.

Mash et al. (64) recruited an extension to the sample of Schauder et al. (80) and found no significant difference between NTD and ASD in cIA (β = 0.031, *p* = 0.412). The multiple regression analysis in ASD showed significant conditional main effects of age and intelligence quotient (IQ). In the regression analyses with the NTD sample, the only significant predictor of cIA was the heart rate. When only participants with IQ ≥ 115 were included in the analysis, age was not related to cIA. When IQ < 115, the relationship between age and cIA was significantly positive in NTD and negative in ASD.

Trimmer et al. (82) investigated SCR when feeling ostracized [cyberball test setup, Williams and Jarvis (90)] in ASD and NTD. Those with ASD showed significantly higher SCR levels in the exclusion condition (*p* < 0.001, ƞ^2^ = 0.36), in contrast to the inclusion condition (*p* = 0.122). In general, the ASD group showed a higher mean level of physiological arousal than the NTD group.

Noel et al. (28) measured IA via a dichotomy cardiovisual simultaneity judgment task in ASD and NTD (cardiovisual simultaneously with different stimuli onset asynchronies, between 0 and 400 ms). In individuals with ASD, cardiovisual temporal acuity was weakly pronounced—cardiovisual temporal binding windows (TBWs) were four times larger (*p* = 0.09) in ASD than in NTD (*p* < 0.001)—the ASD group reports of cardiovisual synchrony appeared nearly independent of stimuli onset asynchronies. Individuals with ASD showed considerably less (did not differ from coincidence) IA than NTD in distinguishing whether their heartbeat was synchronous or asynchronous to the visual stimuli, so that their judgment was almost completely unrelated to whether or not cardiovisual synchrony was present. Participants with ASD showed lower tendencies to report cardiovisual synchrony compared to NTD in total. Noel et al. (28) only compared the actual TBW sizes, and did not report group differences. A mixed-model ANOVA found a clear stimuli × group interaction (*p* < 0.001).

Failla et al. (60) measured cIA (20) during fMRI. There were no significant differences between ASD and NTD regarding IA (*p* = 0.354) or neural (insular) response during the Schandry task (*p* ≥ 0.243).

Tomasi and Volkow (72) extracted the rfMRI datasets of the Autism Brain Imaging Data Exchange I and II database. The thalamic local functional connectivity density in men with ASD revealed lower and weaker age-related increases compared to NTD. There was also a greater right lateralization of Inter-hemispheric Functional Connectivity Density in the inferior parietal cortex in ASD compared with the male NTD sample, but the extreme group comparison was not significant (*p* = 0.06). They found significantly stronger connectivity of the somatosensory, motor, auditory, and interoceptive cortices with the thalamic cluster in the ASD compared to the NTD sample.

Larkin et al. (63) measured IS via the BAQ (36) in older adolescents and adults. The sample contained three groups (based on self-reports): diagnosed with ASD, suspected ASD, and NTD, which did not differ significantly in IS (*p* = 0.078, *η^2p^* = 0.03). IS showed a small negative relationship with severity of ASD (*r* = −0.17, *p* < 0.05).

Wood et al. (52) surveyed IS via the APQ (Autonomic Perception Questionnaire) (91) in individuals with ASD. Social anxiety was positively correlated with IS (*r =* 0.612, *p* < 0.001) in ASD. Higher IS correlated significantly with both higher state self-focused attention (measured by self-rating) and trait self-focused attention (measured via the public subscale) (*r*s ≥ 0.345, *p*s ≤ 0.002). IS mediates the association between ratings of self-performance and social anxiety in ASD.

### 3.4 Meta-analysis: cIA in adults with ASD

#### 3.4.1 Study characteristics

We meta-analyzed the cIA outcomes of adults with ASD and NTD across five studies (60, 66, 67, 71, 79, 81). The underlying data pool is freely available as part of open science in the **Supplementary Materials.** The sample descriptions and each result are shown in **Table 4**. The five studies included *N* = 132 individuals with ASD with mean ages from 25.9 to 37.2 years, and *N* = 134 NTD with mean ages from 25.4 to 41.2 years. In the ASD sample, 74.42% were male, and in the NTD sample, 70.93% were male, with all studies including more men than women. One study did not report participants’ gender. The severity of ASD was measured via AQ (89). The AQ score in individuals with ASD ranged from 31.1 to 35.45.

**Table 4:** Adult studies.

Study		*N*; group	*Age: M (SD)*	*N* females	Age-range	Key findings heartbeat perception	Key sensibility findings (via PBPQ, MAIA or SCR)	Key awareness findings
(74)		N (ASD) = 18 N (NTD) = 18	*M (SD)* = 34.6 (13.3) *M (SD)* = 35.0 (12.8)	0 0	19-60 22-63	–	–	–
(73)		N (ASD) = 16 N (NTD) = 16	*M (SD)* = 34.8 (13.3) *M (SD)* = 36.2 (13.0)	4 7	21–60 23–63	–	–	–
(75)		N (ASD) = 74 N (NTD) = 228	*M (SD)* = 36.7 (12.5) *M (SD)* = 31.5 (11.9)	38 175	18-65 17-67	–	–	ASD<NTD** (BAQ; Shields et al., 1989); ASD<NTD**(TAS, Thirst Awareness Scale)
(77)		N (ASD) = 15 N (NTD) = 15	*M (SD)* = 26.2 (6.4) *M (SD)* = 26.8 (7.8)	0 Not reported	Not reported Not reported	–	ASD>NTD SCR related to empathic pain; ASD<NTD* SCR all images /nonspecific	ASD<NTD empathetic pain discriminability
(88)		N (ASD) = 20 N (NTD) = 20	*M (SD)* = 28.1 (8.8) *M (SD)* = 27.8 (3.4)	2 2	Not reported Not reported	ASD<NTD* (tracking) ASD<NTD Not significant (discrimination)	PBPQ: ASD>NTD*	ASD=NTD
(71)		N (ASD) = 19 N (NTD) = 19	*M (SD)* = 32.9 (11.5) *M (SD)* = 32.9 (14.4)	4 6	Not reported Not reported	ASD=NTD (tracking)	–	–
(81)		N (ASD) = 20 N (NTD) = 20	*M (SD)* = 32.7 (11.2) *M (SD)* = 34.1 (14.2)	3 6	Not reported Not reported	ASD=NTD (tracking)	–	–
(76)		N (ASD) = 13 N (NTD) = 13	*M (SD)* = 38.8 (11.9) *M (SD)* = 40.8 (10.9)	1 0	25–62 19–57	–	ASD=NTD reliable correlations between subj. arousal and SCR	–
(78)		N (ASD) = 17 N (NTD) = 17	*M (SD)* = 26.2 (6.4) *M (SD)* = 26.8 (7.8)	0 Not reported	Not reported Not reported	–	–	–
(66)		N (ASD) = 26 N (NTD) = 26	*M (SD)* = 25.9 (7.3) *M (SD)* = 25.4 (7.6)	7 7	Not reported Not reported	ASD<NTD* (tracking) ASD=NTD (discrimination)	ASD<NTD* (MAIA)	–
(79)		N (ASD) = 46 N (NTD) = 48 N (total) = 94	Not reported Not reported Not reported	Not reported Not reported Not reported	Not reported Not reported 20-64	ASD=NTD (tracking)	–	–
(65)		N (ASD) = 22 N (NTD) = 29 N (total) = 51	*M (SD)* = 27.0 (9.0) *M (SD)* = 27.2 (6.7) *M (SD)* = 27.1	8 13 21	Not reported Not reported 18-53	–	ASD=NTD (MAIA)	–
(61)		N (ASD) = 20 N (NTD) = 20	*M (SD)* = 35.0 *M (SD)* = 34.0	11 11	20-57 22-51	no group difference results available	PBPQ: no group difference results available MAIA: no group difference results available	Measured regarding heartbeat discrimination: no group difference results available
(67)		N (ASD) = 21 N (NTD) = 21	*M (SD)* = 37.2 (11.9) *M (SD)* = 41.2 (14.0)	8 6	24-64 24-65	ASD=NTD (tracking)	–	–
(51)		N (ASD) = 30 N (NTD) = 45	*M (SD) = 39.7 (12.2)* *M (SD) = 35.4 (11.8)*	14 17	*>17* *>17*	–	–	

#### 3.4.2 Fixed-effects meta-regression model

A weighted, fixed-effects meta-regression model tested the association between ASD severity and cIA. As concluded from the raw data, we found no significant correlation between cIA and AQ scores in adults by point as well as interval estimators of *p* (AQ scores as regression weight for cIA being the criterion variable in this case [*r =* −2.75, *p* = 0.39, 95% CI (−9.04, 3.54)]). The pooled effect of the studies is $\hat{\mu }$ = −0.21 (SE = 0.11), with *p* = 0.06 (95% CI: −0.43 to 0.01). There was no significant group difference in cIA measured on the HTT across all studies in adults. Only the extreme edge of the confidence interval indicates significance.

#### 3.4.3 Heterogeneity assessment

The meta-analysis revealed non-significant heterogeneity across included studies. Cochran’s *Q* test indicated homogeneous effect sizes [*Q* (df = 4) = 3.06, *p* = 0.55 > 0.05], suggesting that observed variation between studies was consistent with sampling error alone. *I* ^2^ = 4.61% indicates low heterogeneity (<25% = low heterogeneity). The *H* ^2^ of *H* ^2^ = 1.05 further confirmed minimal excess variability beyond what would be expected from sampling variation. The estimated amount of total heterogeneity is approximately τ^2^< 0.01 (SE: 0.433).

#### 3.4.4 Visual diagnostic assessment

**Figure 2:**
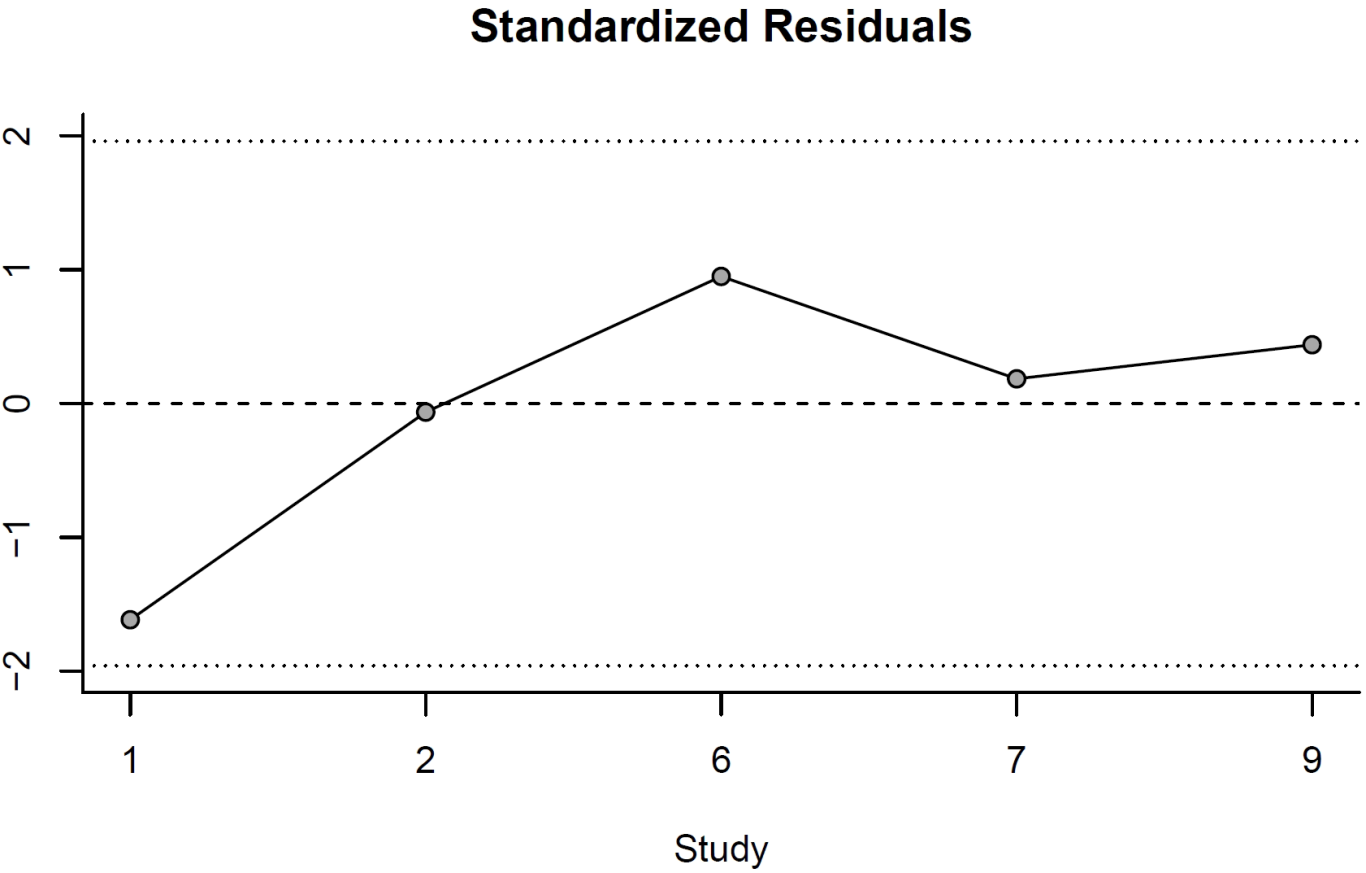
Standardized Residuals.

In order to rule out bias and to check our systematic approach transparently between study effects, the externally standardized residual for the included studies is shown in **Figure 2**. The observed effect size from the pool is shown as residuals. This standardized residuals plot (**Figure 2**) shows that most studies fall within the expected range of ±1.96, with all five studies showing standardized residuals exceeding this threshold. This pattern indicates that the fixed-effects model adequately captures the underlying effect size for most studies, with minimal outlying observations.

**Figure 3:**
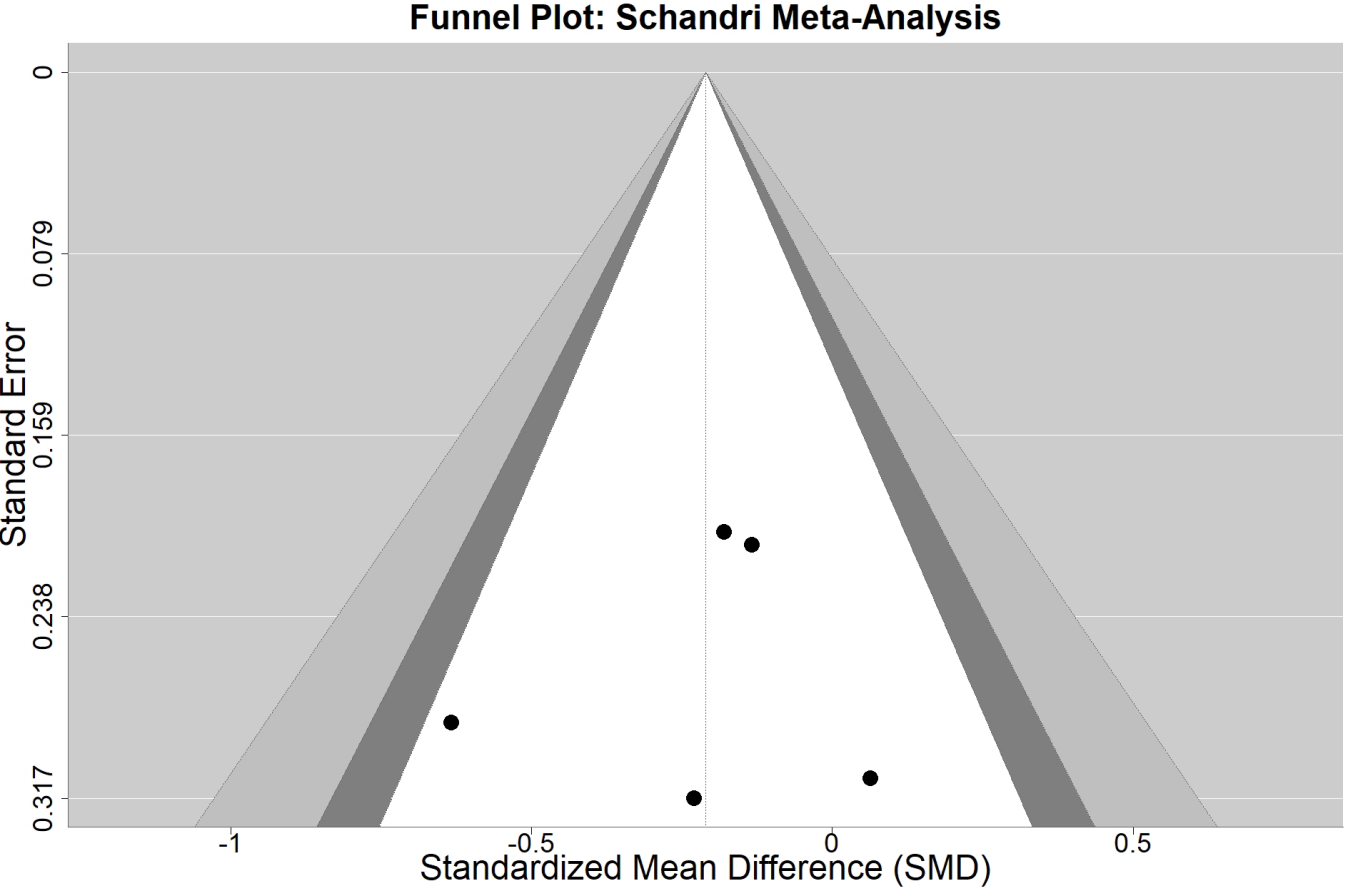
Funnel Plot.

The funnel plot (**Figure 3**) demonstrates a relatively symmetric distribution of effect sizes around the pooled estimate. The majority of studies cluster near the top of the funnel (indicating higher precision), with some scatter at the bottom reflecting the natural increase in variability among smaller studies. The absence of substantial asymmetry suggests minimal evidence of publication bias or small-study effects, supporting the validity of our pooled effect size estimate.

#### 3.4.5 Clinical and statistical implications

The non-significant heterogeneity supports the use of a fixed-effects model and suggests that the included studies are estimating a common underlying effect size. This homogeneity indicates that differences in study characteristics (e.g., sample demographics and measurement approaches) did not substantially moderate the relationship between ASDs and IA. The consistent pattern across studies strengthens confidence in the generalizability of our findings across different populations and methodological approaches.

## 4 Discussion

The primary aim of this review was to systematically summarize and evaluate the current empirical findings regarding a possible association between alterations in interoception and ASD. The review considered studies across the entire lifespan and finally included 31 articles, mainly with a medium (40.6%) or low (also 40.6%) quality. Fourteen studies calculated group differences between an ASD sample and NTD according to the distinguishable dimensions of interoception by Garfinkel et al. (19). There was little evidence of significant and systematic differences between ASD and NTD regarding the studied facets of interoception.

Regarding cIA in children and adolescents: Of seven studies, five (71.4%) concluded that there were no significant group differences in cIA in childhood and adolescents. In contrast, in two studies (28.6%), the ASD sample showed significantly lower cIA in the heartbeat perception task.

Four of the 11 studies in children and adolescents measured IS via the BPQ (32). Two found significantly lower IS in their ASD sample compared to NTD, and two found no significant difference. In summary, the number of results regarding IS does not seem sufficient to draw a definite conclusion regarding possible group differences.

Regarding findings in adults: Of eight publications, nine studies considered group differences in the different cIA tasks. In line with the quantitative results, the face validity by six of the studies conclude that there is no significant group difference in cIA in adults. In contrast, two studies showed significantly lower cIA in ASD, possibly due to variables such as age, gender, or IQ. Mul et al. (66) found significantly reduced cIA in the Schandry task but not in the heartbeat discrimination task. This could mean that the choice and specific implementation of the measurement instrument could affect the results. In summary, there is a fundamental inconsistency in the results. Yang et al. (17) assume that the overall difference in the results of the studies is caused by a wider range of age, as well as cultural and ethnic differences. Number of trials, periods of times, calculation systems, and instructions vary between heartbeat perception tasks. The results in adulthood appear not very different from those in childhood and adolescence. Mash et al. (64) found that cIA decreased significantly with age in the ASD group (in contrast to the NTD). One possible interpretation could be that with increasing age, more external input is taken into account and thus the duration of internal attention decreases. Schauder et al. (80) found that when they compared the shortest time interval in the heartbeat detection task with the longest, the children and adolescents in the ASD sample showed constant cIA compared to NTD. They concluded that individuals with ASD focus their attention on internal sensations for a longer time, or more constantly (80). This could be caused by a longer attention span in general or a preference for heartbeat perception. Regarding this, Mash et al. (64) referred to positive feedback theory, which states that internal stimuli lose salience over time.

In studies of children and adolescents as well as adults, there are conflicting results concerning IS. While Mul et al. (66), using the MAIA for assessing IS, concluded that sensibility is significantly reduced in adults with ASD, Garfinkel et al. (88) concluded that cIA is significantly higher in the ASD sample compared to NTD, when using the BPQ for assessing IS (32). There are several possible reasons for these differences (in the latter case, especially methodological differences). First is the fact that ASD is a diagnosis with high variability or a broad spectrum of pathology (92). The categorical diagnosis ranges from Asperger’s autism or so-called high-functioning autism to high levels of disability, resulting in samples with many individual variations. Approximately 70% of individuals with ASD have a comorbidity with intellectual disability (93), which could also explain the diversity of interoceptive outcomes in ASD. The differences in the results could be caused by different sample characteristics in the studies. Participants differ in their individual constellation of ASD symptoms, their comorbidities, their intellectual impairments, and the severity of their disorder in general. A higher degree of ASD symptoms could result in a lower ability to detect interoceptive bodily signals. The diversity of results could also be explained by different degrees of ASD severity. This could explain why less cIA was found in studies with participants who participated in neurobehavioral treatments prior to testing, as shown by Garfinkel et al. (88).

Some results suggest a developmental perspective, as between-group differences in cIA are found only in children, but not in adults (64, 67). Currently, there are no published longitudinal studies of interoception in ASD (68). Longitudinal designs would need to consider the appropriate time interval between measurements and possible practice effects in testing procedures. Difficulties in perceiving bodily signals in individuals with ASD could subside or be compensated for in adulthood (67). Cognitive abilities could moderate the influence of difficulties in detecting bodily perception signals (64). Investigating interoceptive development seems promising for advancing models of mental disorders and developing innovative interventions. Statistical approaches differ across studies, as in the handling of participant exclusion and statistical outliers.

Particularly in studies of IS, methodological differences could explain the inconsistencies. Palser et al. (68) assumed reduced IS in autistic adults and children, when IS was measured with instruments based on mindfulness, such as the MAIA (35). The interpretation of bodily sensations or mindfulness could be the link to these findings, rather than reduced IS. Introspective ability, response tendencies, and severity of impairment, especially in socio-emotional skills, could also play a role. This is because interaction with other people seems to affect categorization, evaluation, interpretation, and thus the experience of interoceptive signals. Here, observations of responses to pain, to bodily discomfort, and model learning in general are worth mentioning. It seems promising to look at the severity of particularly social and affective domains or restricted and repetitive behavioral domains, rather than just severity overall (68). In recent years, empirical evidence has accumulated, which demonstrates that interoception plays a key role in affect and emotional processes (e.g., 2, 24, 94, 95). Theories that emphasize the importance of interoceptive signals in the development and regulation of emotions, such as embodiment theories and predictive coding perspectives, suggest that socio-emotional difficulties may be due to altered interoceptive perception (68). In this respect, interoceptive difficulties may be equivalent to emotion regulation deficits or may moderate or mediate the association with ASD.

### 4.1 Limitations

Assessments for ASD differ in their conception. The “gold standard” Autism Diagnostic Observation Schedule (ADOS) was not often used in the studies reported here. The AQ test is a popular screening tool, but it has been criticized as not being a reliable predictor of symptoms of ASD (96). Loureiro et al. (57) argue that individuals with ASD challenge with self-evaluation so that their results might not be a reliable indicator due to limited introspection. Most studies in our review used self-reports or questionnaires to capture IS. In the HTT, participants can also be considered as good performers if they have prior knowledge or experience of average heart rate and can estimate heartbeats well. The cardiovascular Signal Detection Task is a new instrument to measure the heartbeat perception task, which compensates for prior knowledge or experience of heartbeat prediction and could improve the accuracy of studies in the future. cIA was usually calculated for each trial to differentiate the measured and the reported heartbeats.

The calculations for the accuracy score differ partially. Since the pre-registration of this systematic review and meta-analysis via OSF, two publications (56, 97) with similar research questions and methodical approaches have been published. The present work differs from these studies in that we assessed strict inclusion criteria, specific questions, and study quality (based on 59). In addition, a confirmed ASD diagnosis was required, no ASD cutoff scores were accepted, and our stepwise testing was performed in a conservative manner.

### 4.2 Conclusion

In conclusion, the results indicate that cIA as measured by heartbeat perception does not seem to be altered in ASD. Regarding cIA in children and adolescents, the majority of studies concluded that there were no significant group differences, while a minority reported significantly lower cIA in the ASD sample. In adults, the majority of publications consistently indicated no significant group differences in cIA across various tasks.

The insular, a hub for interoceptive processing, showed conflicting activation patterns across studies. While increased anterior insular activity during empathy-for-pain tasks aligns with heightened physiological arousal (SCR), reduced insular–amygdala connectivity may underlie difficulties in linking bodily states to emotional experiences. This dissociation mirrors behavioral findings where intact cIA coexists with altered IS in ASD.

The insular’s role in predictive coding offers a framework for these discrepancies: atypical insular responses during pain anticipation could reflect impaired anticipation of bodily states, even when momentary IA remains unaffected. Furthermore, compensatory thalamocortical connectivity patterns might mask insular-specific deficits in resting-state studies. Future research should disentangle task-dependent insular contributions (e.g., during dynamic emotional vs. static interoceptive tasks) to clarify its role in ASD.

## 5 Implications for future research

For future longitudinal research, we suggest standardizing the linguistic age-appropriate adaptations of the test and questionnaires so that meta-analyses are not confronted with different operationalizations of the measurement instruments in future. The same applies to the concrete duration of implementation as well as the evaluation and reporting of the results. Standardization in that regard is urgently required. It would be useful to record which developmental stages have been successfully completed and to what extent developmental delays, for example, in bladder control, are present. Because of different linguistic differentiation and opportunities to communicate, it should be ensured that the individuals with ASD included in studies understand the instructions equally well and can express interoceptive perception verbally.

## Data Availability

The original contributions presented in the study are included in the article/**Supplementary Material**. Further inquiries can be directed to the corresponding author.
