# Supplementary material for: Interoception in individuals with autism spectrum disorder: a systematic literature review and meta-analysis
Source: Front Psychiatry. 2025 Aug 20;16:1573263. doi: 10.3389/fpsyt.2025.1573263 (PMC12406136; doi:10.3389/fpsyt.2025.1573263)
Supplement: Supplementary file 1 [file SupplementaryFile1.zip › Data Sheet 1.pdf]

Supplementary Material

Quality ratings for studies included in the systematic review

Authors	Design	Type of dependent variables	Sample size	Recruitment mode	Use of validated instruments	Comparison groups	Demographic match of control group	Inclusion exclusion criteria	Quality of statistics	Quality assess-ments	Total quality
		**self-report, experimental or multilevel (>one method)**	**based on effect size considera t-ion[a]**	**consecutive sampling (yes/no)**	**for primary/ secondary outcomes (yes/no)**	**none, healthy controls (HC), other controls (OC)**	**demographic match: age and sex (yes/no)**	**provided information sufficient (yes/no)**	**inferential (yes/no)[b]**	**"yes" ratings**	**high, medium, low, unacceptable[c]**
Bird et al., 2010	cross-sectional, quasi-experimental	multilevel	small	yes	yes	NTD	yes	yes	yes	5/5	low (small sample size)
Bernhardt et al., 2014	cross-sectional, quasi-experimental	multilevel	small	yes	yes	NTD	yes	no	yes	4/5	low (small sample size)
Butera et al., 2022	cross-sectional, quasi-experimental	questionnaires	moder ate	no	yes	NTD	yes	yes	yes	3/5	medium
Ebisch et al., 2011	cross-sectional, quasi-experimental	experimental, fMRI	small	yes	yes	NTD	yes	yes	yes	5/5	low (small sample size)
Failla et al., 2020	cross-sectional, quasi-experimental	experimental, fMRI	moder ate	yes	yes	NTD	yes	yes	yes	5/5	high
Fiene & Brownlow, 2015	cross-sectional, quasi-experimental	self-report	large	Not stated clearly	yes	NTD	no	no	yes	2/5	low
Gaigg et al., 2018	cross-sectional, quasi-experimental	multilevel	very small	yes	yes	NTD	no	no	yes	3/5	low (very small sample size)
Garfinkel et al., 2016	cross-sectional, quasi-experimental	multilevel	small	yes	yes	NTD	yes	no	yes	4/5	medium
Gu et al., 2015	cross-sectional, quasi-experimental	multilevel	small	yes	yes	NTD	yes	yes	yes	5/5	low (small sample size)

Gu et al., 2018	cross-sectional, quasi-experimental	multilevel	small	yes	yes	NTD	no	yes	yes	4/5	low (small sample size)
Schauder et al., 2015	cross-sectional, quasi-experimental	multilevel	small	not stated clearly	yes	NTD	no	yes	yes	3/5	low (small sample size)
Shah, Catmur, & Bird, 2016	cross-sectional, quasi-experimental	multilevel	small	not stated clearly	yes	NTD	yes	yes	yes	4/5	low (small sample size)
Shah et al., 2016	cross-sectional, quasi-experimental	multilevel	small	yes	yes	NTD	yes	no	yes	4/5	medium
Larkin et al., 2022	cross-sectional, quasi-experimental	questionnaires	moder ate	not stated clearly	yes	NTD	yes	no	yes	3/5	medium
Mash et al., 2017	cross-sectional, quasi-experimental	experimental	moder ate	yes	yes	NTD	no	yes	yes	4/5	medium (moderate sample size)
Mul et al., 2018	cross-sectional, quasi-experimental	multilevel	moder ate	yes	yes	NTD	yes	yes	yes	5/5	medium (moderate sample size)
Mul et al., 2019	cross-sectional, quasi-experimental	multilevel	small	yes	yes	NTD	yes	no	yes	4/5	medium
Mulcahy et al., 2019	cross-sectional, quasi-experimental	multilevel	large	yes	yes	NTD	yes	no	yes	4/5	high
Nicholson et al., 2018	cross-sectional, quasi-experimental	multilevel	moder ate	yes	yes	NTD	yes	no	yes	4/5	medium
Nicholson et al., 2019	cross-sectional, quasi-experimental	multilevel	small	yes	yes	NTD	yes	no	yes	4/5	low (small sample size)
Nisticò et al., 2022	cross-sectional, quasi-experimental	online-questionnaires	moder ate	yes	yes	NTD	yes	yes	yes	5/5	medium

Noel et al., 2018	cross-sectional, quasi-experimental	experimental	small	yes	no	NTD	no	no	yes	2/5	low
Palser et al., 2018	cross-sectional, quasi-experimental	multilevel	large	yes	yes	NTD	yes	yes	yes	5/5	high
Palser et al., 2020	cross-sectional, quasi-experimental	multilevel	moderate	yes	yes	none	no	no	yes	3/5	medium
Palser et al., 2021	cross-sectional, quasi-experimental	multilevel	moderate	not stated clearly	yes	NTD	no	yes	yes	3/5	medium
Pickard et al., 2020	cross-sectional, quasi-experimental	multilevel	moderate	yes	yes	NTD	no	yes	yes	4/5	medium
Tomasi & Volkow, 2019	cross-sectional, quasi-experimental	experimental	large	no	no	NTD	no	no	yes	1/5	low
Trimmer et al., 2017	cross-sectional, quasi-experimental	multilevel	small	yes	yes	NTD	yes	no	yes	4/5	low (small sample size)
Wood et al., 2022	cross-sectional, quasi-experimental	multilevel	large	yes	yes	none	no	yes	yes	4/5	high
Yang et al., 2022)	cross-sectional, quasi-experimental	multilevel	moderate	no	yes	NTD	yes	yes	yes	4/5	medium
Zdankiewicz-Ścigała et al., 2021	cross-sectional, quasi-experimental	questionnaires	large	no	yes	NTD	yes	yes	yes	4/5	high

[a] Each group: very small < 15 participants, small < 26 participants, moderate 26–63 participants, and large ≥ 64 (Brown & Reuber, 2016; Cohen, 1988).
[b] Inferential (e.g., models controlling for potential confounders; information for probability distribution provided), as opposed to descriptive/explorative.
[c] The quality was assessed as high with ≥ 80% judging "yes" and a good sample size, as medium with 50-79% judging "yes" and at least moderate sample size (or ≥ 80% and moderate sample size), as low with 20-49% judging "yes" or low sample size and as unacceptable when ≤ 20% or a very poor sample size (Brown & Reuber, 2016).
fMRI=functional magnetic resonance imaging, NTD=neurotypically developed.
